# Facilitating collaborative animal research: The development and implementation of a Master Reciprocal Institutional Agreement for Animal Care and Use

**DOI:** 10.1017/cts.2019.431

**Published:** 2020-02-26

**Authors:** Kathryn Holthaus, David Goldberg, Carolyn Connelly, Brian Corning, Christina Nascimento, Elizabeth Witte, Barbara E. Bierer

**Affiliations:** 1 Research Operations, Brigham and Women’s Hospital, Boston, MA, USA; 2 Research Operations, Boston Children’s Hospital, Boston, MA, USA; 3 Broad Institute of MIT and Harvard, Cambridge, MA, USA; 4 Harvard Center for Comparative Medicine, Harvard Medical School, Boston, MA, USA; 5 Harvard Catalyst | The Harvard Clinical and Translational Science Center, Harvard Medical School, Boston, MA, USA; 6 Division of Global Health Equity, Department of Medicine, Brigham and Women’s Hospital, Boston, MA, USA; 7 Department of Medicine, Harvard Medical School, Boston, MA, USA

**Keywords:** Animal care and use, regulatory oversight, IACUC, multi-site review, veterinary care, compliance

## Abstract

Ensuring appropriate review, approval, and oversight of research involving animals becomes increasingly complex when researchers collaborate across multiple sites. In these situations, it is important that the division of responsibilities is clear and that all involved parties share a common understanding. The National Institutes of Health Office of Laboratory Animal Welfare and the United States Department of Agriculture Animal Plant Health Inspection Service require an Institutional Animal Care and Use Committee (IACUC) to review the care and use of animals in research, and both agree that it is acceptable for one IACUC to review the work taking place at multiple institutions. With this in mind, several Harvard-affiliated hospitals and academic centers developed the Master Reciprocal Institutional Agreement for Animal Care and Use (Master IACUC Agreement) to support collaboration, decrease administrative burden, increase efficiencies, reduce duplicative efforts, and ensure appropriate protections for animals used in research. Locally, the Master IACUC Agreement has fostered greater collaboration and exchange while ensuring appropriate review and oversight of research involving animals. As multisite animal protocols become more prevalent, this Agreement could provide a model for a distributed, national network of IACUC reliance.

## Introduction

Institutions have an overarching responsibility to ensure appropriate review, approval, and oversight of research involving animals. Additional complexity emerges when institutions must oversee research that is occurring at or otherwise involves multiple institutions. As scientists increasingly collaborate across multiple sites – working with researchers locally and nationally – the task of ensuring research oversight becomes increasingly complex. In these situations, the division of responsibilities between and among institutions must be clear and involved parties should share a common understanding. This is particularly relevant as animal regulations differ by funding agency and individual institutional policies are variable.

Both the National Institutes of Health Office of Laboratory Animal Welfare (OLAW) and the United States Department of Agriculture (USDA) Animal Plant Health Inspection Service (APHIS) require an Institutional Animal Care and Use Committee (IACUC) to review the care and use of animals in research and determine whether proposed research projects are in accordance with federal policy [1–3], regardless of whether the work occurs at one or multiple institutions. However, OLAW and the USDA-APHIS also agree that the regulations do not require review of a research project by more than one IACUC [4]; it is acceptable for one IACUC to review the work taking place at multiple institutions.

With that in mind, several collaborating Harvard-affiliated hospitals and academic centers sought to address this gap by developing a common reciprocal agreement that would support collaboration, decrease administrative burden, increase efficiencies, reduce duplicative efforts, and, importantly, ensure appropriate protections for animals used in research. Spearheaded by Harvard Catalyst | The Harvard Clinical and Translational Science Center, the resulting Master Reciprocal Institutional Agreement for Animal Care and Use (Master IACUC Agreement) was jointly drafted by a cohort of compliance officers and IACUC directors who represent the 18 original signatory institutions^1^ and was executed. The Master IACUC Agreement outlines reciprocal and administrative oversight capacity relating to functions and activities of the institutions’ IACUCs and is intended to maintain and enhance institutional effectiveness while avoiding duplication of efforts.

**Fig. 1.**
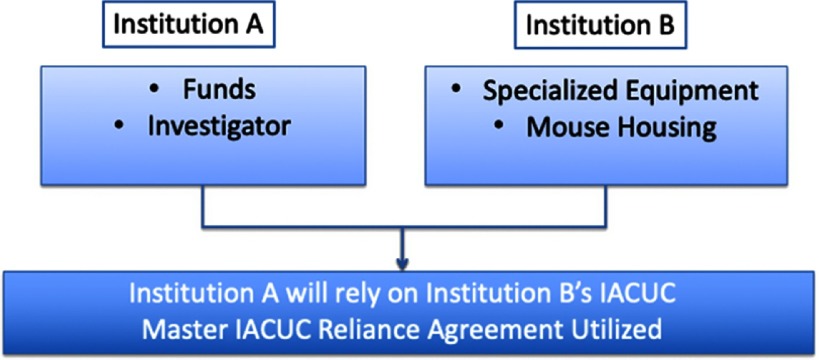
Use of Master Institutional Animal Care and Use Committee (IACUC) Reliance Agreement for Animal Work Performed Off-site.

**Fig. 2.**
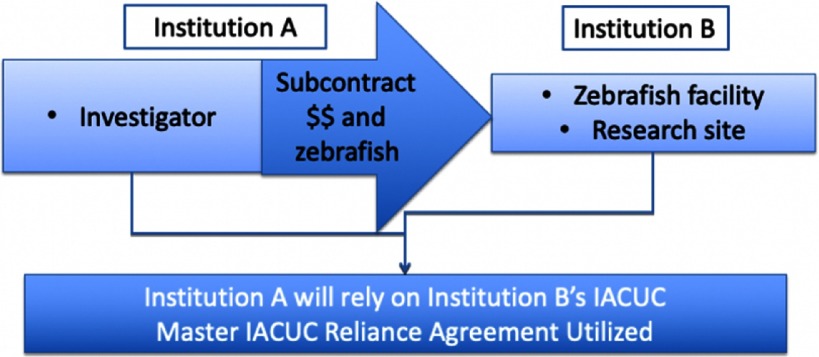
Use of Master Institutional Animal Care and Use Committee (IACUC) Reliance Agreement in Subcontract Work.

The Master IACUC Agreement covers responsibilities for the care, use, ownership, transport, and transfer of all vertebrate animals and addresses regulations set forth by OLAW, USDA-APHIS (if applicable), and other regulatory agencies (e.g., Department of Defense), as well as standards set by AAALAC International. With execution of this agreement, signatory institutions can avoid the time- and resource-intensive process of drafting and executing individual, project-specific agreements. Moreover, the exercise of drafting the Master IACUC Agreement provided the opportunity for the animal research oversight groups at each signatory institution to jointly discuss best practices and align to common expectations, while also having the added benefit of building upon their existing relationships to further future collaboration. As part of the drafting process, cases were presented and “what if” scenarios worked through (e.g., see Figs. 1 and 2), ensuring the resulting agreement’s broad utility.

In this example, the research involves animal activities in one location that are funded by a different institution at a different site. By utilizing the Master IACUC Agreement, one institution (e.g., the institution receiving the funds, Institution A) may choose to defer review to the other (usually the one doing the animal work, Institution B). All aspects of the experiment will take place inside Institution B’s animal facility, which will also house the mice.

In this example, the researcher at Institution A is outsourcing the animal work to another signatory institution, Institution B, with the appropriate expertise and facilities for the animal model. By utilizing the Master IACUC Agreement, one institution may choose to perform the IACUC review and provides oversight for the research, so that administrative burden for the researcher as well as the institution is reduced.

## Methods and Approach

In the USA, a number of federal regulations govern the use of animals in research. The Animal Welfare Act (AWA) and Animal Welfare Act Regulations dictate the care and use of animals in many areas, including research. US institutions must have an IACUC to review and approve, or withhold approval of, every protocol and research project that involves animals. The IACUC plays a major role in ensuring that research animals are used responsibly and are cared for in a humane manner. First adopted in 1966, the AWA covers all warm-blooded animals except rats (of the Genus *Rattus*), mice (of the Genus *Mus*), and birds bred for research. The AWA further excludes certain farm animals. APHIS oversees AWA compliance. Additionally, research institutions that receive support through the US Public Health Service (PHS) or the National Science Foundation for research involving animals must also comply with the PHS Policy on Humane Care and Use of Laboratory Animals [1], requiring institutions to have an IACUC to review protocols and the animal program, among other oversight functions. Unlike the AWA, PHS Policy oversees all vertebrate animals, including laboratory-bred rats, mice, birds, and species such as fish and frogs; the PHS Policy is overseen by OLAW. Finally, institutions may also be subject to state and local regulations as well as institutional policies. Each institution establishes policies and procedures to describe the local implementation of the regulations.

As noted above, OLAW and USDA-APHIS agree that review of a research project by more than one IACUC is not a federal requirement [5]. The National Research Council’s *Guide for the Care and Use of Laboratory Animals* (the *Guide*) [6] in 2011 established expectations for inter-institutional research collaborations involving animals, noting the potential for ambiguities in oversight responsibilities, protocol review, animal care and use, and ownership. It made clear that institutions should have a formal documentation (e.g., a contract, memorandum of understanding (MOU), or agreement) addressing the responsibilities for offsite animal care and use, animal ownership, and IACUC review and oversight [7]. While OLAW offers an inter-institutional assurance template, the brief one-page document is utilized for a different and limited purpose: when one institution does not have its own IACUC, animal facilities, or animal care and use program and will instead conduct activities using live vertebrate animals at another institution that does have an OLAW-approved assurance. There is no template delineating responsibility among collaborating institutions that each has an OLAW-approved assurance. In the absence of a comprehensive template or more detailed guidance, these institutions are left to determine how to operationalize the oversight of animal care and use in multi-institutional research projects.

Familiar with the challenges of collaboration, representatives from the IACUCs at Harvard’s schools and affiliated institutions recognized the potential benefits of a master IACUC reliance agreement. HMS sits among affiliated schools and hospitals in Boston and Cambridge, Massachusetts. Collaboration is challenging. Despite a common affiliation, these legally independent entities each have their own institutional policies, interpretations of how best to operationalize and comply with federal policies, and methods of oversight for animal research. Without adequate oversight and clarity, collaborations that cross institutions may raise biosecurity and compliance concerns. By detailing the terms of a reliant IACUC review arrangement, the cohort of compliance officers and IACUC directors who came together to develop the Master IACUC Agreement sought to alleviate these concerns among the area institutions and eliminate the burden of project-specific negotiations.

This effort was modeled, to a degree, on the Harvard Catalyst Master Reciprocal Common Institutional Review Board (IRB) Reliance Agreement, first executed in 2009, that established the framework, substantive legal provisions, and operational elements essential to provide institutions a flexible alternative to duplicative IRB review and support collaborative human subjects research [8, 9]. The IACUC reliance agreement sought to establish a similar model for the review and oversight of animal research.

Representatives from three Harvard-affiliated institutions and Harvard Catalyst drafted the Master IACUC Agreement. This core group met first to describe current operations and oversite functions of collaborating institutions. The institutions agreed upon animal ownership and responsibility for animal care, but initially had varying views of IACUC and compliance responsibilities, including reporting responsibilities (e.g., when collaborating institutions should be notified of noncompliance, which institution should notify oversight agencies) and the frequency of protocol updates (e.g., triennial, amendments) to the collaborating institutions. Further, the representatives debated which institution should perform congruency review for studies funded by the federal government prior to release of funds. The group came to consensus through identification of best practices, benchmarking with others, and discussion. Finally, a draft MOU authorization template was developed that included an initial set of assurances, elements, and responsibilities.

Once the MOU template was complete, the core group worked through various “use cases” of potential collaborative scenarios. Examples of these scenarios include animal work performed at an institution that differs from a collaborating institution that is funding the work (Fig. 1), animal work subcontracted to another signatory institution with the expertise or facilities to perform the work (Fig. 2), collaborating institutions sharing a joint animal protocol that prefer to subject the protocol to only one review, animals tested in one location and then transferred for a procedure to a second institution, and many others. The MOU template was revised to address these use cases that were then used to explain the utility of the MOU agreement to other IACUCs, Institutional Officials, and legal counsel (see Supplemental Material).

The master IACUC reliance template was reviewed by each institution’s IACUC and legal counsel. The draft was then distributed to an additional four institutions and the larger local IACUC community for feedback; suggestions were incorporated, and revisions made. The final draft agreement was sent to OLAW for feedback and to ensure that the draft was concordant with the PHS requirements. The final iteration was again locally distributed and revised, and the final Master IACUC Agreement was reviewed and signed by the Institutional Officials.

## Terms of Agreement, Roles, Responsibilities

The drafting institutions addressed several key decision points regarding the agreement’s scope, applicability, and assignation of responsibilities. The Agreement sets the terms and conditions by which one site may rely on another to discharge its animal care and use responsibilities; the Agreement does not, however, mandate or make automatic any reliance. Each institution retains the right to choose on a case-by-case, protocol-by-protocol basis whether to rely or perform its own IACUC review.

The group determined that each signatory institution must maintain an Animal Welfare Assurance with OLAW as well as other applicable permits or registrations with state and municipal agencies (e.g., the Massachusetts Department of Public Health, Boston Public Health Commission). Additionally, each must maintain its own USDA registration, if housing species regulated by USDA. Institutions that are or become accredited by AAALAC International are required to meet AAALAC standards; non-accredited institutions use the National Research Council’s *Guide* as the basis for developing and implementing an institutional program for animal activities. Each signatory institution designates a contact person or liaison who will communicate on behalf of the institution with respect to the Agreement.

The development of the Agreement necessitated the alignment and clarification of key responsibilities related to IACUC review and oversight (see Table 1) including transport of the animals. With regard to possession and transport, the signatory institution in possession of the animals in question assumes ownership. If animals are transported to another signatory institution, the responsibilities for the animals remain with the originating signatory institution until receipt of the animals by the receiving institution. The signatory institution where the actual activities and review are conducted, known as the “Performance Site,” is responsible for animal care and use, including veterinary oversight. The institutions agreed that, while not required by OLAW, the Performance Site is best positioned to perform the IACUC review and address oversight requirements. The institution that is receiving the prime award to conduct the animal work but will rely on another institution for review, housing, or performance of the animal work is termed the “Relying Site.”

**Table 1.** Key elements of the Master Institutional Animal Care and Use Committee (IACUC) Agreement

Eligibility requirements	Existence and maintenance of an Animal Welfare Assurance^a^ Existence and maintenance of United States Department of Agriculture (USDA) registration, if working with a USDA-covered species Maintenance of or commitment to meet the standards required for accreditation from the Association for Assessment and Accreditation of Laboratory Animal Care (AAALAC)
Responsibilities	Ownership: The institution in possession of the animals assumes ownership, unless otherwise agreed upon in advance Congruence between animal protocol and research grant or contract: the institution granted the award is responsible for grant-protocol congruence (may rely on the performance site for the congruency review) Protocol Review: The Performance Site is responsible for ensuring that animal care and use complies with Public Health Service (PHS) Policy, the Animal Welfare Act, the Guide for Care and Use, institutional policies and other applicable laws, statutes, and guidance, as appropriate Rights of institutions: An appropriate institutional representative in a given collaboration may choose to: attend IACUC meeting at institution for protocol review visit the space within which animals are housed or used request minutes of protocol review or semi-annual reports include the site in a post-approval monitoring audit/program
Documentation, Notification, and Reporting	Performance Site is responsible for compliance with regulatory requirements, including maintaining required documentation; reporting to accrediting, federal, state, and local agencies; and providing this information to the Relying Site upon request (e.g., protocol documents or approvals, significant deficiencies, reports of non-compliance, USDA inspection reports) Institution receiving grant, contract, or award is responsible for financial regulatory requirements (i.e., reporting non-compliance to funder) Performance Site must inform relying site(s) of loss or suspension of Office of Laboratory Animal Welfare (OLAW) assurance, USDA registration, or AAALAC accreditation Each institution must submit its own annual reports to OLAW, USDA, AAALAC, and any other regulatory or oversight organizations An institution in receipt of a FOIA request must forward that request to the other institution participating in any research protocol that is the subject of or impacted by the request

a https://olaw.nih.gov/guidance/topic-index/animal-welfare.htm.

While the Performance Site reviews and approves a protocol, a Relying Site may, upon request, send a representative to attend the relevant portion of the IACUC meeting; alternatively, the Performance Site may require the Relying Site to send a representative to the IACUC meeting. Relevant sections of the meeting minutes are made available to Relying Sites upon request. Additionally, Relying Sites may request to inspect the Performance Site’s facilities.

As noted above, the intent of the Agreement is to reduce duplicative review and encourage collaboration while also respecting institutional autonomy. Reliance on another site’s review is therefore not required, and each site reserves the right to conduct its own review through its own IACUC. In such cases, where multiple IACUC reviews will occur for a single study, the Agreement responsibilities note that the relevant signatory institutions will conduct such reviews in consultation and collaboration to ensure congruency of approved protocols. While each institution retains responsibility for grant congruency review, if multiple institutions are included on one grant, the participating institutions may agree in advance that a single institution is best suited to perform this congruency review. That institution will then notify the other sites, once the analysis is complete, of any identified discrepancies and their resolution.

The designated reviewing IACUC, the Performance Site, follows written procedures for reporting to the Relying Site: notification of approval by the reviewing IACUC; any relevant dates and any protocol stipulations; the approval of the initial protocol; approval of amendments to research activities; approval of continuing review of research (annual reviews); review and reporting of unexpected events or incidents or significant issues related to animal welfare reported in the semi-annual report; and other findings and actions of the designated reviewing IACUC that affect the proposed animal work.

The Performance Site will report any significant deficiencies identified during facility or program review and related to the projects covered by the Agreement to the Relying Sites. The Performance Site is also responsible for investigating noncompliance or adverse events; a Relying Site may also request that the Performance Site conduct a for-cause audit or the Relying Site may conduct its own investigation, working collaboratively with the Performance Site to implement corrective actions. The Performance Site assumes responsibility for review and reporting of incidents to any relevant oversight agencies (e.g., OLAW, USDA, AAALAC, etc.) and shall notify the Relying Sites should such reporting be necessary. The Performance Site is responsible for any suspension, disapproval, or termination of the activities, and for notification of the Relying Site(s) should such action be required. Relying Sites agree that they may not override or reverse such a decision by the Performance Site.

A Performance Site must advise Relying Sites should any change occur in the status of the site’s PHS, AWA, or USDA registration, or any loss of AAALAC accreditation, or if it is under investigation by USDA or OLAW. Likewise, the Performance Site will notify Relying Sites of any findings related to the reliance activities.

All signatory institutions are required to maintain records in accordance with federal regulations, and each is responsible for submitting its own annual reports to USDA, OLAW, and AAALAC, as required. Each Performance Site (i.e., the reviewing IACUC) is responsible for reporting for those protocols under their oversight. USDA-regulated species, however, are included in the USDA Annual Report (at the highest pain category reached) of the institution where they are housed, regardless of where procedures are performed.

The Agreement also includes provisions regarding requests received under the Freedom of Information Act (FOIA). Upon notification by a federal agency or department of a FOIA request, the signatory institution will forward the notice to all other signatory institutions participating in the relevant research activities. The notified signatory institution will respond to the FOIA notice after soliciting, from each participating institution, suggestions for the application of FOIA exceptions, including but not limited to seeking extension(s) of time to respond.

In the event a public signatory institution receives a request under a state public records law, the institution shall forward it to all other participating signatory institutions. All participating institutions cooperate in responding to the request and asserting exceptions to disclosure of information, if applicable. Non-FOIA or non-state public records law request is handled in a similar manner.

As a master agreement, the participating institutions sought to allow for expansion of the Agreement; therefore, the Agreement includes a joinder process through which new signatories could agree to the terms of the Agreement without requiring re-signature for all parties. A similar process was already in use for the Harvard Catalyst Master IRB Reliance Agreement. Thus, any new institution that meets the eligibility conditions may sign a Joinder Agreement accepting the terms and conditions of the master Agreement. In general, eligibility is contingent on: (1) consideration of participation by existing signatory institutions; (2) active and current Animal Welfare Assurance with OLAW and, if housing species regulated by the USDA, USDA registration; and (3) designation of a contact-person or liaison who will communicate on behalf of the institution with respect to the Agreement.

Any signatory may terminate its participation in the Agreement upon a 90-day advance written notice to the other signatory institutions, and the affected institution(s) will work together to determine and minimize the effect of termination on any ongoing reliance activities at the time of termination.

## Discussion

The Master IACUC Agreement is responsive to regulatory expectations for the review and oversight of research activities involving the care and use of animals. While aiming to address these expectations in a clear and defined manner, the Agreement itself notes that it may not cover every possible situation involving animal research. Matters arising that are not addressed by the Agreement are addressed in good faith between the parties. The legal document and framework delineate roles and responsibilities, define decision making authority, and allow reliance to be determined voluntarily, on a study-by-study basis. The detailed delineation of responsibilities differs significantly from the previously available OLAW inter-institutional assurance; moreover, as a *master* agreement, the Master IACUC Agreement includes multiple institutions and eliminates the need for repeated renegotiation and signature of study-specific agreements. The upfront discussions and negotiations among the stakeholder institutions did take some time, albeit significantly less time than if each agreement was negotiated individually. Additionally, these conversations allowed for candid discussions of best practices and provided confidence that all participating institutions were operating under the same interpretations of the guidance documents, policies, and regulations.

The Master IACUC Agreement has been used to facilitate collaborations, while assuring regulatory compliance and outlining specifically the distribution of responsibilities. While many of the signatory institutions share a collegial relationship, the Agreement has fostered greater collaboration and exchange. The signatory institutions have been inviting one another to attend IACUC meetings regularly and have shared best practices, policies, and trends within their programs. Developing the Agreement led to the expansion of a local, “Greater Boston” IACUC Consortium that convenes, as needed, to discuss changes in and implementation of federal regulations. The ongoing close relationships between the institutions foster a sense of collaboration among not only the researchers but among the IACUCs as well.

The Agreement has been particularly helpful in reporting of noncompliance, since the terms and specifics of responsibilities were outlined prior to any noncompliance event. All signatories respect confidentiality and routinely work together to ensure any reporting is accurate, transparent, and complete. Further, in those situations where noncompliance is discovered, the collaborating IACUC Director routinely informs their counterpart directly and shares a copy of the draft report to federal oversight agencies in advance of the actual submission. Allowing adequate time for discussion, review, and revision ensures that each institution is adequately represented and fosters further good will.

Signatory institutions have received positive feedback regarding the Agreement during AAALAC site visits and USDA annual inspections. In particular, when questions have arisen during inspections, the Agreement is of great utility as it clearly articulates the housing location of animals and the assignation of IACUC review and reporting responsibilities.

The Agreement is flexible by design and includes a process for amendments to the Agreement itself. Following the initial Master Agreement, three institutions created guidance to clarify the review process for congruency between the protocol and grant or award. This guidance was then proposed to the signatories via an amendment to the Master IACUC Reliance Agreement. The amendment allowed non-prime awardee institutions the ability to perform the congruency review and provided guidance on responsibilities and communication. Although, both the amendment and the process for the adoption of the amendment worked well, there have been no additional amendments to the Agreement. It would be challenging if a proposed amendment failed to obtain agreement among signatories thus necessitating tracking of who has signed and who abstained from adopting the amendment, particularly because one of the many benefits of this agreement is the predictability and concordance of processes among institutions that collaborate frequently.

Many institutions may benefit from a similar arrangement, and the agreement itself allows for additional institutions to join. The joinder process allows institutions to become part of the Reliance Agreement, immediately sharing in the benefit of – but not mandating – reliance. While it may introduce some complications, the Master IACUC Agreement could be adapted for use internationally; however, in its present form, the Agreement requires signatory institutions to follow only those regulations and policies relevant to research in the USA. If all participating international institutions are AAALAC accredited, the agreement could be augmented to include only reference to AAALAC standards, at the participating institutions discretion.

The Agreement is based upon voluntary collaboration; its success depends upon competent and capable performance, experience, and effective communication. As multisite animal protocols become more common, the Master IACUC Reliance Agreement could become the model for collaboration and a distributed network of reliance, similar to the SMART IRB model for human participant research. Greater cooperation, clarity of responsibilities, and exchange of beneficial information will advance science while maintaining and improving the ethical and humane treatment of animals that is foundational to research.

## Footnotes

1 Beth Israel Deaconess Medical Center; Boston Children’s Hospital; Brigham and Women’s Hospital; Broad Institute; Dana-Farber Cancer Institute; Forsyth Institute; Harvard Medical School (HMS); Harvard T.H. Chan School of Public Health; Harvard School of Dental Medicine; Harvard University Faculty of Arts and Sciences (FAS); Joslin Diabetes Center; Massachusetts Eye and Ear; Massachusetts General Hospital; Massachusetts Institute of Technology; McLean Hospital; MGH Institute of Health Professions; Schepens Eye Research Institute; Spaulding Rehabilitation Hospital.

## Supplementary material

For supplementary material accompanying this paper visit http://dx.doi.org/10.1017/cts.2019.431. click here to view supplementary material
