# Supplementary material for: Facilitating collaborative animal research: The development and implementation of a Master Reciprocal Institutional Agreement for Animal Care and Use
Source: J Clin Transl Sci. 2020 Feb 26;4(2):96–101. doi: 10.1017/cts.2019.431 (PMC7159813; doi:10.1017/cts.2019.431)
Supplement: Supplementary file 1 [file S205986611900431Xsup001.pdf]

Master Reciprocal Institutional Agreement for Animal Care and Use

A. Purpose

Each institution, operating under its own authority, has specific responsibilities for fostering proper animal care and welfare. The many ethical and logistical issues that arise from the use of live vertebrate animals in research are further complicated when research involves the collaboration of two or more institutions. This Master Reciprocal Institutional Agreement for Animal Care and Use (the "Agreement") sets forth understandings of reciprocal and administrative oversight capacity relating to functions and activities of the institutions' Institutional Animal Care and Use Committees ("IACUCs") and is intended to maintain and enhance institutional effectiveness while avoiding duplication of efforts.

B. Agreement Scope and Definitions

This Agreement by and between or among all of the institutions signing below (the "Signatory Institutions"), addresses living vertebrate animal ownership and responsibility for animal care and use, IACUC review, and IACUC oversight when funded at one or more of the Signatory Institutions or collaborative work with living vertebrates for research, teaching or testing and other collaborative activities occurs at more than one Signatory Institution. The "Performance Site" is the Signatory Institution where the actual animal care and use activities and IACUC review are conducted. A "Relying Site" is a Signatory Institution that (1) proposes to engage in Animal Reliance Activity with a Performance Site; (2) is required by law or policy to conduct its own IACUC review and oversight of the activities in question; and (3) elects to cede these functions to the Performance Site. An "Animal Reliance Activity" is any activity that involves the care and use of animals at a Performance Site in collaboration with one or more Reliance Sites. Animal Reliance Activities may include (but are not limited to) the following activities:

- Transfer of animals from Relying Site to the Performance Site for conduct of research, testing or education; or
- Transfer of animals from Relying Site to the Performance Site for housing and care; or
- Relying Site staff participation in research involving the care and use of animals at the Performance Site; or
- A Relying Site's payment for animal work conducted at the Performance Site, but no animals are transferred and no Relying Site personnel are directly involved; or
- A Relying Site's collaboration with Performance Site for scientific purposes, where Performance Site hosts the animal component of a collaborative work.

This Agreement sets the terms and conditions by which a Relying Site may rely on a Performance Site's discharge of its animal care and use responsibilities but does not mandate or make automatic any reliance. Each Signatory Institution retains the right to choose on a case-by-case basis whether to rely under this Agreement or perform its own IACUC review. Relevant records and/or documentation for the scope of work and any payment or funding for each individual protocol or project utilizing this Agreement will be created and maintained individually by the Signatory Institutions participating in the research according to the standards set by those institutions.

C. Signatory Institution Requirements

Each Signatory Institution hereby shall maintain an Animal Welfare Assurance with the Office of Laboratory Animal Welfare ("OLAW") of the National Institutes of Health under the jurisdiction of the Public Health Service ("PHS") as well as other applicable permits or registrations with state and municipal agencies (e.g., the Massachusetts Department of Public Health, Boston Public Health Commission). Each Signatory Institution housing species regulated by the United States Department of Agriculture ("USDA") hereby shall also maintain its own USDA registration. Signatory Institutions that are accredited or become accredited by the Association for Assessment and Accreditation of Laboratory Animal Care International ("AAALAC") shall meet AAALAC standards; non-accredited institutions shall use the National Research Council's *Guide for the Care and Use of Laboratory Animals* (the "*Guide*") as a basis for developing and implementing an institutional program for activities involving animals.

Each Signatory Institution shall designate a contact-person or liaison who will communicate on behalf of the Signatory Institution with respect to matters concerning implementation of this Agreement, and shall notify the other Signatory Institutions promptly if there are changes in the contact-person.

D. Responsibilities

1. Unless otherwise agreed in advance, the Signatory Institution in possession of the animals involved in the Animal Reliance Activities assumes ownership of the animals. During transport:

- When a Signatory Institution directly transports animals (i.e., without use of a third-party courier), the institution transporting the animal(s) is responsible for animal care and use in transit and for reporting any non-compliance or animal welfare issues that arise in the course of transporting them.
- When a third-party courier is used for transport, the originating Signatory Institution is responsible for the care and use of animals in transit and for reporting, in accordance with the terms of this Agreement, any non-compliance or animal welfare issues that arise until the receiving Signatory Institution receives the animals.

Once the receiving Signatory Institution is in possession of the animals, the receiving Signatory Institution is then responsible for care and use of the animals as well as for reporting, in accordance with the terms of this Agreement, any non-compliance or animal welfare issues that arise,

2. The Performance Site is responsible for animal care and use, including veterinary oversight.

3. Care and use of animals will be conducted by Performance Site in compliance with all laws and regulations, including the Animal Welfare Act and its regulations (9 C.F.R. pt. 2) (together the "AWA"), if applicable the PHS Policy on Humane Care and Use of Laboratory Animals (the "PHS Policy"), and the most recent edition of the *Guide.*

4. Care and use of animals regulated by the USDA will be conducted only if the Performance Site is registered with the USDA, as required by law.

5. The Performance Site shall review and, as appropriate, approve all animal protocols that fall within the scope of this Agreement.

6. A Relying Site, with notice, may send a representative to attend that portion of the Performance Site's IACUC meeting where a Relying Site's protocol or non-compliance will be reviewed. In addition, the Performance Site may require a Relying Site to send a representative to the Performance Site's IACUC meeting. Relevant portions of Performance Site meeting minutes will be made available to a Relying Site upon written request.

7. A Signatory Institution reserves the right to conduct its own review of animal care and use of Animal Reliance Activity at a Performance Site through its IACUC. The Signatory Institution's duplicative review should be done in consultation and collaboration with the Performance Site so that the two resulting, approved protocols are congruent. The Signatory Institutions agree that it is their desire to conduct duplicative review only in rare circumstances.

8. Each Signatory Institution will be responsible for conducting the grant-to-protocol congruency comparison for funding awarded to their institution, regardless of which institution is the Performance Site, according to the standards set by their institution and the PHS Policy. However, institutions may agree in advance who is best suited to do the congruency review. The institution that conducts the congruency analysis will notify the other when the analysis is completed. To the extent an approved protocol is not congruent with its corresponding grant, the institution conducting the congruency analysis will communicate this to the other and together the institutions will cooperate in addressing the discrepancy issues, including, but not necessarily limited to, requiring an amendment to the protocol.

9. Regardless of the source of funding for the care and use of animals, the Relying Site(s) may, upon reasonable notice and during regular business hours, inspect any of the Performance Site's facilities where Animal Reliance Activities are or have been conducted. The Relying Site(s) shall abide by all policies, procedures and directions of the Performance Site while on Performance Site premises.

E. Documentation, Notification and Reporting

1. The Performance Site shall provide the following documentation, notification, and reporting, as applicable, to Relying Site(s):

> a. Upon request, the Performance Site will provide a copy of the IACUC-approved protocol, any IACUC-approved amendments and any corresponding IACUC approval letters to a Relying Site as soon as possible after IACUC approval, but no later than 30 days from IACUC approval.

b. Upon request, the Performance Site will provide documentation relevant to the use or care of animals governed by this Agreement including but not limited to the animal protocol, relevant portions of the program review, as well as documentation related to facility inspection.

c. If any significant deficiencies relevant to an Animal Reliance Activity are noted during the semi-annual facility or program review, a Performance Site shall report such relevant significant deficiencies along with the plans for correction, in writing, to all Relying Sites within 5 business days of a request for correction. For significant deficiencies representing serious reportable animal health and welfare issues, a Performance Site shall report to the affected Relying Site(s) promptly and within no more than 2 business days. Upon request, the Performance Site shall provide to the Relying Site(s) documentation of the Performance Site's approval for the initial or most recent triennial review as well as any subsequent amendments or annual review (if USDA-covered species) of Animal Reliance Activity protocol.

d. The Performance Site is responsible for investigating non-compliance issues or adverse events. The Relying Site may request that the Performance Site conduct a for-cause audit and may choose to accept the findings of the Performance Site, or a Relying Site may conduct its own investigation into Animal Reliance Activities at the Performance Site. If a Relying Site elects to perform its own investigation, it will make every effort to work collaboratively with the Performance Site on implementation of corrective actions.

e. In accordance with institutional requirements, a Performance Site shall assume responsibility for review and federal reporting of any incidents of non-compliance with or any significant deficiencies under PHS Policy, the *Guide*, or AWA. For incidents of non-compliance or significant deficiencies in connection with Animal Reliance Activities, the Performance Site shall provide a corrective action plan to the affected Relying Site(s). If the Performance Site determines that its findings in connection with Animal Reliance Activities are reportable to OLAW, USDA, and/or other oversight entities, it shall so notify the Relying Site(s). The Performance Site shall notify OLAW, USDA and/or other oversight entities, as applicable. Once the Relying Site(s) receives such notification, Relying Site is responsible for reporting to any applicable funding agency or funding sponsors. At a minimum, the Performance Site shall copy Relying Site(s) on any reports of non-compliance to federal agencies in connection with Animal Reliance Activities and shall provide Relying Site(s) with received copies of all replies.

f. In accordance with institutional requirements, after identifying a significant deficiency or incident of non-compliance in an Animal Reliance Activity, a Performance Site is responsible for any suspension, disapproval or termination of the Animal Reliance Activity. The Performance Site shall notify applicable Relying Site(s) immediately in writing if it suspends, disapproves or terminates an Animal Reliance Activity. Relying Site(s) acknowledge and agree that they may not override or reverse a decision by the Performance Site to suspend or terminate IACUC approval.

g. A Performance Site shall within 5 business days advise the Relying Site(s) of any change of status in its PHS Animal Welfare Assurance or USDA registration, any loss of AAALAC Accreditation, if applicable, or any notification that the Performance Site is under investigation by the USDA and/or OLAW. In limited situations the use of a confidentiality agreement may be necessary and considered.

h. Following inspections by any outside agency (such as USDA, OLAW or AAALAC), a Performance Site shall notify Relying Site(s) of any findings specifically pertaining to Animal Reliance Activities promptly, and generally within 5 business days of receipt. In limited situations the use of a confidentiality agreement may be necessary and considered.

2. Maintenance of records for Signatory Institutions shall be in accordance with federal regulations governing record retention policies for PHS and USDA.

3. Each Signatory Institution will be responsible for submitting its own annual report(s) to OLAW, USDA and AAALAC. Information regarding protocols for which the institution is designated as the Performance Site/IACUC will be included in the Performance Site's report. USDA-regulated species will be included in the USDA Annual Report of the institution where they are housed, regardless of where procedures on them are performed.

4. In the event a federal agency or department notifies a Signatory Institution that the agency or department has received a request under the Freedom of Information Act ("FOIA") for information on Animal Reliance Activities subject to this Agreement, the Signatory Institution receiving the FOIA notice shall forward it to all other Signatory Institutions participating in the Animal Reliance Activities identified in the underlying FOIA request. The Signatory Institution will take the lead in responding to the FOIA notice and will endeavor to solicit from each participating institution that institution's suggestions for the application of FOIA exceptions, including but not limited to seeking extension(s) of time

from the government to provide feedback so that all affected participating institutions will have a reasonable time to comment.

In the event a public Signatory Institution receives a request under a state public records law for information on Animal Reliance Activities subject to this Agreement, the Signatory Institution shall forward it to all other Signatory Institutions participating in the Animal Reliance Activities identified in the request. All participating institutions, including the Signatory Institution, shall cooperate in responding to the request and asserting exceptions to disclosure of information, if applicable.

If the Signatory Institution receives a non-FOIA or non-state public records law request, it shall be handled in the same manner as outlined for FOIA requests.

If the Signatory Institution receives no response from a Signatory Institution about a FOIA, state public records law request, or a non-FOIA or non-state public records law request that request disclosure about another Signatory Institution, it shall, if it has not taken this action already, obtain an extension of time to respond to the government, if applicable, and send a second notice to the participating institution about the need to respond. If after two notices, the participating institution still fails to respond, the Signatory Institution is hereby authorized to assert all appropriate exceptions, if applicable, as the Signatory Institution deems applicable

F. Miscellaneous

1. Signatory Institutions acknowledge that this Agreement may not have covered every possible situation involving animal research. Matters arising that are not addressed by the Agreement will be addressed in good faith between the parties and, if applicable, among the Signatory Institutions.

2. This Agreement may be modified by mutual written consent of all of the Signatory Institutions.

3. Any Signatory Institution may terminate its participation in the Agreement upon a 90-day advance written notice to the other Signatory Institutions. In the event of any termination, the affected Signatory Institution(s) will work together to determine and minimize the effect of such termination on any ongoing Animal Reliance Activities at the time of termination.

4. This Agreement does not supersede any prior agreements between or among the Signatory Institutions. Signatory Institutions shall remain free to contract with one another for matters affecting their respective animal care programs.

5. No inferences about any Signatory Institution or its program shall be drawn simply on the basis of its participation in this Agreement, and no Signatory Institution shall be responsible for the acts or omissions of other Signatory Institutions simply by virtue of the fact that all are parties to the Agreement.

G. Joinder Process

This Agreement is open to participation by any additional institution (each a “New Institution”) that meets the eligibility conditions specified below and signs a Joinder Agreement, in the form attached hereto as Exhibit A, by which the New Institution accepts the terms and conditions of this Agreement and any subsequent amendments then in effect.

In general, a New Institution’s eligibility for participation in this Agreement is contingent on:

- Consideration of participation by existing Signatory Institutions; and
- Active and current Animal Welfare Assurance with OLAW and, if housing species regulated by the USDA, USDA registration; and
- Designation of a contact-person or liaison who will communicate on behalf of the New Institution with respect to matters concerning implementation of this Agreement, and notifying the other Signatory Institutions promptly if there are changes in the contact-person.

Each current Signatory Institution acknowledges and agrees that if New Institution meets the criteria listed above, it will be accepted to join this Agreement.

H. Signatory Institutions

Execution of this Agreement provides assurance that the review and continuing oversight by the Relying Site(s) and the Performance Site(s) meets the animal subject protection requirements of that institution's OLAW-approved Animal Welfare Assurance, USDA registration and AAALAC accreditation, if applicable. The Institutional Officials signing below agree to accept and rely on the review and approval of Animal Reliance Activities by each Signatory Institution acting as a Performance Site in accordance with the agreed terms and conditions, without the need for additional institutional signatures. All Signatory Institutions must keep this document on file.

Not all Signatory Institutions must sign to make this Agreement effective. This Agreement is effective immediately as between or among the Signatory Institutions as of the date each signed. This Agreement may be executed in one or more counterparts, each of which shall be deemed an original but all of which together shall constitute one and the same instrument. Facsimile or other electronic signatures may be deemed originals.

EXECUTED BY AUTHORIZED PARTICIPATING INSTITUTIONAL OFFICIALS

Institution 1

Animal Welfare Assurance #:<br>USDA Registration #:<br>AAALAC Unit #:<br>AAALAC accreditation date:	______________________________<br>Signature<br>Printed Name:<br>Title:<br>Date:

Institution 2

Animal Welfare Assurance #:<br>USDA Registration #:<br>AAALAC Unit #:<br>AAALAC accreditation date:	______________________________<br>Signature<br>Printed Name:<br>Title:<br>Date:

Institution 3

Animal Welfare Assurance #:<br>USDA Registration #:<br>AAALAC Unit #<br>AAALAC accreditation date:	______________________________<br>Signature<br>Printed Name:<br>Title:<br>Date:

Institution 4

Animal Welfare Assurance #:<br>USDA Registration #:<br>AAALAC Unit #:<br>AAALAC accreditation date:	______________________________<br>Signature<br>Printed Name:<br>Title:<br>Date:

Institution 5

Animal Welfare Assurance #:<br>USDA Registration #:<br>AAALAC Unit #:<br>AAALAC accreditation date:	______________________________<br>Signature<br>Printed Name:<br>Title:<br>Date:

Institution 6

Animal Welfare Assurance #:
USDA Registration #:
AAALAC Unit #:
AAALAC accreditation date:

____
Signature
Printed Name:
Title:
Date:

Institution 7

Animal Welfare Assurance #:
USDA Registration
AAALAC Unit #:
AAALAC accreditation date:

____
Signature
Printed Name:
Title:
Date:

Institution 8

Animal Welfare Assurance #:
USDA Registration #:
AAALAC Unit #:
AAALAC accreditation date:

____
Signature
Printed Name:
Title:
Date:

Exhibit A

Form Joinder Agreement for the Master Reciprocal Institutional Agreement for Animal Care and Use

NAME OF INSTITUITON

Animal Welfare Assurance #:<br>USDA Registration #:<br>AAALAC Unit #:<br>AAALAC accreditation date:	______________________________________<br>Signature<br>Printed Name:<br>Title:<br>Date:

This Joinder Agreement, which is effective as of the date written above (the "Effective Date") is intended to establish that _________________________ ("New Institution") has become a party to the **Master Reciprocal Institutional Agreement for Animal Care and Use** dated as of __________ (the "Agreement").

1. Definitions. Capitalized terms used but not defined herein shall have the meaning set forth in the Agreement.

2. Joinder; Binding Provisions of Agreement. New Institution hereby agrees to join the Agreement and become a Signatory Institution. As a signatory, the New Institution hereby accepts and agrees to be bound by the obligations and to enjoy the rights of Signatory Institutions under all the terms and conditions of the Agreement.

3. Notices. All notices and other communications from any other Signatory Institution made to New Institution concerning the Agreement shall be delivered to the following addresses:

For Notice:

Name of IACUC contact:

Title:

Institution:

Address:

Town, State, Zip Code:

Name of Institutional Official:

Title:

Institution:

Address:

Town, State, Zip Code:
